# Supplementary material for: Exploring Online Health Information Seeking Behaviour (OHISB) among young adults: A scoping review protocol
Source: BMJ Open. 2024 Jan 30;14(1):e074894. doi: 10.1136/bmjopen-2023-074894 (PMC10828883; doi:10.1136/bmjopen-2023-074894)
Supplement: Author's manuscript [file bmjopen-2023-074894.draft_revisions.pdf]

BMJ Open is committed to open peer review. As part of this commitment we make the peer review history of every article we publish publicly available.

When an article is published we post the peer reviewers' comments and the authors' responses online. We also post the versions of the paper that were used during peer review. These are the versions that the peer review comments apply to.

The versions of the paper that follow are the versions that were submitted during the peer review process. They are not the versions of record or the final published versions. They should not be cited or distributed as the published version of this manuscript.

BMJ Open is an open access journal and the full, final, typeset and author-corrected version of record of the manuscript is available on our site with no access controls, subscription charges or pay-per-view fees (http://bmjopen.bmj.com).

If you have any questions on BMJ Open's open peer review process please email info.bmjopen@bmj.com

BMJ Open

Healthy Choices and the Social Gradient WP5 protocol: a scoping review exploring online health information seeking behavior (OHIS) among young adults

Journal:	*BMJ Open*
Manuscript ID	bmjopen-2023-074894
Article Type:	Protocol
Date Submitted by the Author:	19-Apr-2023
Complete List of Authors:	Bratland, Kristine; UiT Norges arktiske universitet, Department of Community Medicine<br>Wien, Charlotte; University of Southern Denmark, Clinical Research<br>Sandanger, Torkjel; UiT Norges arktiske universitet, Department of Community Medicine
Keywords:	Protocols & guidelines < HEALTH SERVICES ADMINISTRATION & MANAGEMENT, SOCIAL MEDICINE, PUBLIC HEALTH, Decision Making

SCHOLARONE™
Manuscripts

Healthy Choices and the Social Gradient WP5 protocol: a scoping review exploring online health information seeking behavior (OHIS) among young adults

Abstract:

Introduction: In recent years, the growth of information technology and social media accelerated at an unprecedented pace, and there was also a substantial increase, especially in the Nordic countries. However, there is a noticeable lack of comprehensive understanding of the latest research findings on online health information seeking behavior (OHIS), particularly among young adults. There is a need for an updated review on knowledge gaps, provide research-based guidance, and recommendations to governments, health organizations, and social media platforms on how to support this prominent pattern. The scoping review protocol describes the systematically mapping of the existing literature on young adults' preferences for digital platforms and platform characteristics in relation to OHIS, allowing identification of promising areas for further research and development of more effective interventions to promote healthy and informed choices. A scoping review is urgently needed to provide a comprehensive understanding of young adults' OHIS, to support the next generation of dissemination that promotes accurate and reliable digital health information.

Methods and analysis: This scoping review will be conducted using Arksey and O'Malley's methodological framework (PRISMA-ScR), employing the Citation Pearl Method and the SPIDER model to design the search strategy. To find relevant literature, three databases will be searched: Scopus, Web of Science, and EMBASE. Additionally, a subsidiarily grey literature search will be conducted in Google Scholar. The data charting process will follow the PRISMA-ScR standard and will be further structured with EndNote. Qualitative and quantitative analyses of the extracted data will be developed using EndNote and Excel.

Ethics and dissemination: Scoping review are secondary data analysis of publicly available sources and do not require an ethical review. The findings will be shared through open access peer-reviewed publications, and with service users and stakeholders in national and international conferences, social media platforms, newspapers, and YouTube.

Strengths and limitations of this study:

- A systematic study conducted following the SPIDER model using EndNote and the PRISMA-ScR checklist.
- A study in a developing and cross-disciplinary research field.

For peer review only

- Limited to include articles published within the last six years.
- Conducted in an underserved and rapidly developing research field.

Introduction

Health information seeking behavior (HISB), as defined by Johnson (1997), was a deliberate attempt to meet a health information need. This distinguished it from passive forms of information acquisition, such as incidental exposure or scanning (Shim, Kelly, & Hornik, 2006). Online health information seeking behavior (OHIS) emerged in the late 1990s and early 2000s, coinciding with the advent of the internet as a promising source of health information (Eysenbach et. al. 2002; Dutta-Bergman, 2004; Galarce et. al. 2011). Research on health information seeking behavior has grown significantly in the last decade, driven by the rapid increase in health information available and the shift from an authoritative clinician-patient interaction to shared decision making (Lewis et. al. 2021).

Research on young adults' online health information seeking behavior is crucial as it can help identify gaps in health knowledge, improve health literacy, and promote health equity (Azzopardi-Muscat et. al. 2019; Sun 2019). Understanding how young adults use digital tools to seek and process health information, and how this information shapes their health behaviors is important. Considering young adults are heavy users of technology, they make a critical audience for online health information (Lewis et. al., 2006; Jensen et. al., 2019). Reviewing the current state of research on OHIS is crucial for informing health communication and intervention strategies due to the potential impact of such information on their health behaviors and outcomes. Such a review is also novel in that it provides a comprehensive synthesis of existing studies, which are often fragmented and lack consistency in conceptualization and measurement of key constructs.

The purpose of conducting a scoping review is to develop a transparent and reproducible method that can be adapted and applied by others in future research. Additionally, a protocol allows for a more rigorous control of the review process, ensuring that the research questions are systematically and comprehensively addressed. The review will investigate online health information seeking behavior (OHIS) to provide an overview of the current state of knowledge and identify gaps in the literature. A comprehensive search of electronic databases, including EMBASE, Scopus, and Web of Science, using relevant keywords related to OHIS will be conducted. Inclusion criteria are established to ensure that only studies meeting predetermined criteria are included in the review (Tricco et al., 2008). The data extraction will involve collecting information on study design, sample characteristics, and key findings related to OHIS (Arksey & O'Malley, 2005). The synthesis of the literature will be analyzed thematically

For peer review only

to identify key themes and sub-themes (Thomas et al., 2008). The scoping review may contribute to the field by identifying areas for future research (Levac et al., 2010), such as the core components of the digital platforms young adults use to search for information and the characteristics of the user interface of the chosen platform. This protocol advocates for using the scoping review methodology for exploring a broad research topic such as OHIS. (Arksey & O'Malley, 2005; Levac et al., 2010).

Methods and analysis

The search strategy was inspired by the scoping review methodology suggested by Arksey and O'Malley (2005) and further developed by Levac, Colquhoun, and O'Brien (2010) and Peters et al. (2015). This methodological approach allowed for a comprehensive search of the literature, identification of relevant studies, and mapping of the extent and nature of the available evidence (Arksey & O'Malley, 2005; Thomas & Harden, 2008; Levac et al., 2010) on online health information seeking behavior among young adults. Using the scoping review approach enables the mapping of existing literature, clarification of key concepts, identification of evidence gaps, and reporting of evidence that can inform decision-makers (Arksey & O'Malley, 2005; Munn et al., 2018). While the methodical framework of Arksey and O'Malley (2005), further developed by Levac et al. (2010), informs the overall conduct of the scoping review, the Preferred Reporting Items for Systematic Reviews and Meta-Analyses (PRISMA) extension for Scoping Reviews' (Tricco et.al. 2018) guides the reporting of this protocol and also subsequently structures the reporting of the full review.

For peer review only

Stage 1: Defining the research question

This protocol aims to describe a possible approach to a scoping review where the assessment of previous research aims to identify which platforms young adults tend to seek out to find health information and which characteristics on the chosen platform(s) appeal to them regarding trustworthiness and readability. The overall purpose of the scoping review is:

What does previous studies report on young adults' online health information seeking behavior (OHIS) on health information regarding the choice of digital platform and platform user interface (UI)?

The review is guided by the following objectives:

1) Identifying studies on young adults' online health information seeking behavior
2) Determining which digital platforms young adults tend to search for health information
3) Identifying characteristics in the user interface that apply to young adults' aims and trust

4) Enhancing the characteristics that have an impact.

Stage 2: Identifying relevant literature

In the scoping review on online health information seeking behavior among young adults, a systematic approach is used to choose the search terms. The following steps are taken: A preliminary search is conducted with the support of subject matter experts in the field to identify additional keywords and search terms. Controlled vocabulary (MeSH-terms) and keywords are used to build the first search string, which results in the identification of three relevant papers. Citation Pearl Growing (Ramer, 2005) is applied by thoroughly reading the three core papers from the preliminary search and searching for new search terms. The bibliography is revisited to look for relevant references published prior to the core documents that could contain other possible search terms. The search string is revisited and developed to ensure that it captures all the relevant concepts related to the research question. The search string is presented to a new group of experts in the field for feedback, and changes are made accordingly. The initial search string is designed using key elements from the review question, applying the SPIDER strategy (Sample, Phenomenon of Interest, Design, Evaluation, Research type). The SPIDER tool is an alternative to the more well-known PICO, better suited when searching for qualitative research papers (Cook et al., 2012), replacing the "O" (Outcome) with the broader term "E" (Evaluation) (Cook et al., 2012).

**Sample**	Young adults in the age range where they make their own choices, meaning over 18, under 30 years old. No limitations in gender. Preferably in the Nordic countries.
**Phenomenon of Interest**	Online Health Information Seeking Behavior
**Design**	Published literature of any research design, grey literature
**Evaluation**	"Searching activities" - characteristics, views, experiences, observations, descriptions, surveys, descriptive
**Research type**	Qualitative, quantitative, mixed methods, multi methods peer reviewed studies. Grey literature including third sector and conference proceedings.

Table 1: The SPIDER tool applied to the review question

**Sample**	("young adults" OR young OR "early adulthood" OR "early twenties" OR "aged 20") AND
**Phenomenon of Interest**	"Health Information" AND
**Evaluation**	AND (search* OR seek* OR find* OR access* OR retrieve* OR behavior*) AND (internet OR online OR web OR digital* OR media)

Table 2: The proposed search strategy mapped against the SPIDER tool

Boolean operators (AND, OR) are utilized to combine the various search terms, ensuring that relevant articles that meet the inclusion criteria are retrieved. Multiple rounds of trials and errors will be conducted. Through these techniques, the search strings are ensured to be comprehensive and capture the relevant concepts (Adams et al., 2017) related to online health information seeking behavior among young adults.

For peer review only

When conducting a scoping review, it is important to choose appropriate databases to ensure that all relevant studies are captured (Arksey & O'Malley, 2005; Peters et al., 2015). To identify relevant articles for the scoping review on online health information seeking behavior among young adults, a comprehensive search is conducted on three electronic databases: EMBASE, Web of Science, and SCOPUS. These three databases are commonly used in health research, and each has its own strengths and weaknesses (Stapleton et. al 2020; Bramer et al., 2016). EMBASE is a biomedical database that covers a wide range of journals, including many that are not indexed in MEDLINE (Frandsen et. al. 2021; Bramer 2018). It includes content related to medicine, nursing and allied health sciences and research, including systematic reviews, making it a valuable resource for researchers and healthcare professionals (Bramer 2016). Web of Science is a multidisciplinary database that covers a wide range of journals in the social sciences, humanities, and sciences. It is particularly strong in its coverage of citation information, making it a useful tool for tracking the impact of research (Norris 2007; Gavel et. al. 2008). SCOPUS is a multidisciplinary database that covers a wide range of journals in the health sciences, social sciences, and sciences. It is particularly strong in its coverage of conference proceedings and grey literature (Godin 2015; Falagas et al., 2008; Gavel 2008). By searching multiple databases, the risk of missing important studies that may be indexed in one database but not in another is reduced. These databases also have advanced search capabilities that allow for refinement of the search strategy and retrieval of only the most relevant studies (Bramer et al., 2016). They are widely used in health research, which means that the review can be compared and contextualized with other studies that have used similar methods and data sources.

After identifying the relevant articles from the databases, the citation pearl method will be used to identify additional relevant studies. This method involves examining the reference lists of the selected articles to identify any other relevant studies that may not have been captured in the initial search (Bramer et al., 2017). A forward citation search will also be conducted to identify any new articles that cite the selected articles since their publication. This will help to identify newer studies that may not have been captured in the initial search or the citation pearl search. The citation pearl method is a commonly used technique in systematic reviews and scoping reviews to ensure that all relevant studies are included in the analysis (Tricco et al., 2018). It is particularly useful when searching for grey literature or when there are no standard indexing terms to identify relevant articles. By using this method, additional studies that meet the inclusion criteria will be identified, ensuring that the scoping review is comprehensive and robust. After the advanced search, a search will be conducted in Google Scholar using the advanced search feature to detect grey literature. Specific search filters will be applied, such as limiting results to conference proceedings, reports, theses, dissertations, and other non-peer-reviewed sources. Furthermore, an advanced search will be conducted for grey literature

For peer review only

using Google Scholar to ensure that no relevant studies are missed (Adams et al., 2019). Grey literature comprises research that is not published in traditional academic journals or other peer-reviewed sources and includes reports, conference proceedings, and dissertations (Godin et al., 2015). It can be difficult to locate such literature through traditional search methods, and it is important to include it in a scoping review to consider all available evidence. By using Google Scholar, our search for grey literature will be broadened beyond traditional academic sources to identify additional studies on online health information seeking behavior among young adults. A combination of keywords and advanced search techniques will be employed to retrieve relevant grey literature (Bramer et al., 2018). This approach will help identify a range of studies that may have been missed in the initial database search. By using traditional academic databases and Google Scholar to search for grey literature, the scoping review on online health information seeking behavior among young adults will be made as comprehensive as possible, including all available evidence on the topic.

Stage 3: Article selection

As part of the scoping review process, researchers will establish inclusion and exclusion criteria based on the research question and topic of interest. Inclusion criteria will include factors such as publication date, study design, population, intervention, and outcomes, while exclusion criteria will include factors such as language, publication type, and study quality (Thomas & Harden, 2008). By carefully planning and executing the inclusion and exclusion of articles, researchers will ensure that their scoping review is comprehensive, relevant, and reliable (Arksey & O'Malley, 2005; Levac et al., 2010).

Criterion	Definition
**Population**	Population of young adults (>18 and ≤30), or explicit address this part of the population. Surveys that also screened children or adults will be included if the procedure for young adults is described.
**Screening procedure**	Peer reviewed publications on a health-related topic with the aim of describing, elaborate, systematize, and/or quantifying online health information seeking behavior, use of internet, search engines, browsers, web 2.0 or 3.0, social media or other online platforms retrieving information.
**OHIS definitions**	A qualitative or quantitative description of an active effort to obtain health-related information about a specific topic, using any digital sources to obtain desired information, that occurs outside of routine patterns of interpersonal communication or media use (Case 2016; Lewis et. al. 2021; Lewis et. al 2006).
**Platform**	Interested in determining what digital platforms young adults tend to use when searching for health information

For peer review only

**Characteristics**	Interested in identifying traits, characteristics, or interface on these digital platforms that young adults prefer, enjoy, or has a significant meaning for those using the exact platform
**Setting**	Primarily observational studies or self-reported studies from the global north. However, may include studies developed in other parts of the world if the methodology employed is subsequently.

Table 3: Inclusion and exclusion criteria

To identify papers about OHIS behavior in young people, a set of inclusion and exclusion criteria is developed. The following will be the criteria for inclusion:

1) The topic of the article must be health-related, or encompassing topics e.g., mental health, diet, or nutrition.
2) The study must target young adults or specifically address the population of older adolescent or make explicit, equivalent assertions. Although the search strategies indicate two commonly accepted lower age boundaries, “early twenties” and “aged 20” to identify young adults above teens, it will not exclude other ways to describe the population.
3) Describe OHIS activities or individual preferences or assessments, e.g., general OHIS, search strategies, choice of health information sources, or use of health information.
4) The paper must be published in 2017 or later, to make the results applicable to today's digital landscape.

The exclusion criteria are as follows:

1) Studies that focus on the use of general information and communication technology or behavior change, instead of focusing on health information will be excluded.
2) Papers where age is a determinant in studying the OHIS of the overall population will be excluded, as it is apparent that age has an influence on individual OHIS behavior. Abstracts, posters, op-eds, or letters will be excluded as they are not full papers.
3) Papers that are not published in a peer-reviewed journal or in the proceedings of a conference will be excluded.
4) Papers written in languages other than English, Norwegian, Swedish, or Danish will be excluded.

When the search is conducted and the articles are retrieved in EndNote, the next step will involve screening them for eligibility based on established criteria. A three-stage screening process will be performed, where researchers will first remove duplicates. The next step will be to screen titles and abstracts to exclude obviously irrelevant articles. This will be followed by a full-text screening of the remaining articles to determine whether they meet the inclusion criteria. Additionally, EndNote will be

For peer review only

used as a citation management software to facilitate the screening process and keep track of the articles throughout the review. Each step of the process will be filed in separate EndNote Libraries. The reasons for excluding articles will be documented, and the progress will be made visible by presenting a PRISMA flow diagram, which will include searches of the databases, registers, and other sources. This will ensure transparency and reproducibility of the review process.

Once the search is conducted and the articles are retrieved and stored in a labeled EndNote-library, the next step will involve screening them for eligibility based on the established criteria. This will involve a two-stage screening process, where titles and abstracts will be first screened to exclude obviously irrelevant articles, followed by a full-text screening of the remaining articles to determine whether they meet the inclusion criteria. The results in each step will be stored in new separated EndNote libraries. This will be important to document the process of excluding articles, to ensure transparency and reproducibility of the review process, and will make the scoping review comprehensive, relevant, and reliable.

Stage 4: Data charting

To record and synthesize the extracted data, a data charting form will be used. The form will help to organize the data into categories and subcategories and identify gaps in the literature. Descriptive statistics such as frequency and percentage will be used to summarize the extracted data. The aim of the scoping review is to provide a comprehensive overview of the existing literature on OHIS in young adults to inform future research and practice in this area. The findings of the review will help to identify current trends, patterns, and factors influencing young adults' OHIS behavior, including the benefits and risks associated with OHIS. The results will also inform the development of strategies to improve the quality and accessibility of online health information for young adults.

Variable	Definition
First author	The last name of the papers first author
Year	The year the paper was published
Title	Title of the article
Journal	Name of the journal
Nationality	Nationality of the publisher/university
Country	Country/nationalities of the population
Sample Size	The number of participants, informants, or observations
Population	Group, sex, age

For peer review only

Method	Type of data and/or tools: Qualitative (survey, interview, administrative data, observation, focus group) quantitative (check list, self-report, chart reviews, electronic health record, validated questionnaire,
Results	The main results or findings
Other notes	Additional information about the paper

Table 4: Variables and definitions for data chartering

Stage 5: Collating, summarizing, and reporting the results

EndNote and Excel will be used to arrange and scrutinize the data extracted from the full-text articles. The goal is to produce both quantitative and qualitative data while analyzing the articles comprehensively to evaluate them and achieve the objectives of the review. Excel will be used to analyze the data generated, and following the PRISMA guidelines (Tricco et al., 2018), assessments of the qualitative and quantitative data will be integrated to present the results. Findings will be tabulated by identifying:

1) Where young adults report finding health information
2) Choice of digital platform
3) Preferences regarding user interface

Expected limitations and strengths of review finding

The upcoming scoping review on online health information seeking behavior among young adults will aim to identify research gaps, map, and synthesize evidence, and summarize findings using a transparent and systematic approach. The study will focus on identifying which digital platforms young adults tend to search for health information, as well as characteristics in the user interface that young adults trust and find useful when seeking health information online. The scoping review will be conducted using the Arksey and O'Malley (2005) framework and the PICO model (da Costa Santos et al., 2007) to develop a search strategy, while Excel will be used to chart and organize the data, and the PRISMA-ScR checklist (Tricco et al., 2018) will guide the reporting of findings. To ensure the quality of the scoping review, the search will be limited to studies published in peer-reviewed journals. However, the heterogeneity of the included studies will be acknowledged to limit the ability to provide a quantitative synthesis of the data, such as a meta-analysis. Instead, a focus will be made on a qualitative synthesis of the evidence, which will provide a comprehensive overview of the research in the field. By synthesizing the evidence, gaps in the literature will be identified, and insights into the characteristics of digital platforms that are most effective for young adults when seeking health

For peer review only

information online will be provided. The findings will be reported in a peer-reviewed journal, following the scoping review reporting standards (PRISMA-ScR) and disseminated widely to stakeholder groups using innovative media approaches.

Ethics and dissemination

Ethical considerations will remain essential in all health research, including scoping reviews. In Norway, the ethical principles for medical research are set out in the Health Research Act (Hforsknl, 2008), which emphasizes the importance of protecting the rights and privacy of research participants. As this scoping review will not involve human research participants, it will not be necessary to obtain informed consent, protect anonymity, or receive institutional board approval. However, we will still ensure the integrity of the research and respect for all potential stakeholders by following established guidelines for conducting scoping reviews, such as the PRISMA-ScR checklist (Tricco et al., 2018) and the Arksey and O'Malley (2005) framework. The findings will be disseminated through various channels, including open access peer-reviewed journal publications, national and international conference presentations, academic social media platforms, newspaper articles, and blog posts. By sharing our results widely, the aim will be to inform and engage a diverse audience, including individuals affiliated with our institutions and professional associations.

For peer review only

References:

Adams, R. J., Smart, P., & Huff, A. S. (2017). Shades of grey: guidelines for working with the grey literature in systematic reviews for management and organizational studies. *International Journal of Management Reviews*, *19*(4), 432-454.

Arksey, H., & O'Malley, L. (2005). Scoping studies: towards a methodological framework. *International journal of social research methodology*, *8*(1), 19-32.

Bramer, W. M., Giustini, D., & Kramer, B. M. (2016). Comparing the coverage, recall, and precision of searches for 120 systematic reviews in Embase, MEDLINE, and Google Scholar: a prospective study. *Systematic reviews*, *5*, 1-7.

Bramer, W. M., Giustini, D., Kleijnen, J., & Franco, O. H. (2018). Searching Embase and MEDLINE by using only major descriptors or title and abstract fields: a prospective exploratory study. *Systematic Reviews*, *7*, 1-8.

Case, D. O., & Given, L. M. (2016). Looking for information: A survey of research on information seeking, needs, and behavior.

Cooke, A., Smith, D., & Booth, A. (2012). Beyond PICO: the SPIDER tool for qualitative evidence synthesis. *Qualitative health research*, *22*(10), 1435-1443.

Dutta-Bergman MJ. (2004) Primary sources of health information: comparisons in the domain of health attitudes, health cognitions, and health behaviors. Health Commun. 16(3):273-88. doi: 10.1207/S15327027HC1603_1. PMID: 15265751.

Eysenbach, G., Powell, J., Kuss, O. and Sa, E. R. 2002. Empirical studies assessing the quality of health information for consumers on the World Wide Web: A systematic review. *Journal of the American Medical Association*, 287: 2691–2700.

Falagas, M. E., Pitsouni, E. I., Malietzis, G. A., & Pappas, G. (2008). Comparison of PubMed, Scopus, web of science, and Google scholar: strengths and weaknesses. *The FASEB journal*, *22*(2), 338-342.

Frandsen, T. F., Eriksen, M. B., Hammer, D. M. G., Christensen, J. B., & Wallin, J. A. (2021). Using Embase as a supplement to PubMed in Cochrane reviews differed across fields. *Journal of Clinical Epidemiology*, *133*, 24-31.

Galarce, E. M., Ramanadhan, S., & Viswanath, K. (2011). Health information seeking. In *The Routledge handbook of health communication* (pp. 194-207). Routledge.

Gavel, Y., & Iselid, L. (2008). Web of Science and Scopus: a journal title overlap study. *Online information review*, *32*(1), 8-21.

For peer review only

Godin, K., Stapleton, J., Kirkpatrick, S. I., Hanning, R. M., & Leatherdale, S. T. (2015). Applying systematic review search methods to the grey literature: a case study examining guidelines for school-based breakfast programs in Canada. *Systematic reviews*, *4*(1), 1-10.

Hforsknl (2009). Lov om medisinsk og helsefaglig forskning (helseforskningsloven). LOV-2008-06-20-44. Available: https://lovdata.no/dokument/NL/lov/2008-06-20-44 [Accessed 9 Feb 2023].

Jensen, M., George, M. J., Russell, M. R., & Odgers, C. L. (2019). Young adolescents' digital technology use and mental health symptoms: Little evidence of longitudinal or daily linkages. *Clinical Psychological Science*, *7*(6), 1416-1433.

Johnson, J. D. 1997. *Cancer-related information seeking*, Cresskill, NJ: Hampton Press.

Lewis, N., Shekter-Porat, N., & Nasir, H. (2021). Health Information Seeking. *The Routledge Handbook of Health Communication*, 399-411.

Levac D, Colquhoun H, O'Brien KK. (2010). Scoping studies: advancing the methodology. Implement Sci 5:69.

Lewis, T. (2006). Seeking health information on the internet: Lifestyle choice or bad attack of cyberchondria?. *Media, Culture & Society*, *28*(4), 521-539.

Lewis, S. P., Mahdy, J. C., Michal, N. J., & Arbuthnott, A. E. (2014). Googling Self-injury: the state of health information obtained through online searches for self-injury. *JAMA pediatrics*, *168*(5), 443-449.

Morgan, E. R., Laing, A., McCarthy, S., & Diaz-Ponce, A. (2017). What young people want from an online health community: a qualitative study. Journal of Medical Internet

Norris, M., & Oppenheim, C. (2007). Comparing alternatives to the Web of Science for coverage of the social sciences' literature. *Journal of informetrics*, *1*(2), 161-169.

Peters M, Godfrey C, McInerney P, et al. JBI reviewer's manual, chapter 11: Scoping reviews: the Joanna Briggs Institute;. Available: https://reviewersmanual.joannabriggs.org/ [Accessed 13 Feb 2023].

Peters MDJ, Godfrey CM, Khalil H, et al. (2015). Guidance for conducting systematic scoping reviews. Int J Evid Based Healthc 13:141–6.

Ramer, S. L. (2005). Site-ation pearl growing: methods and librarianship history and theory. *Journal of the Medical Library Association*, *93*(3), 397.

For peer review only

Shim, M., Kelly, B., & Hornik, R. (2006). Cancer information scanning and seeking behavior is associated with knowledge, lifestyle choices, and screening. *Journal of Health Communication*, *11*(S1), 157-172.

Stapleton, J., Carter, C., & Bredahl, L. (2020). Developing systematic search methods for the library literature: Methods and analysis. *The Journal of Academic Librarianship*, *46*(5), 102190.

Sun, Y., Zhang, Y., Gwizdka, J., & Trace, C. B. (2019). Consumer evaluation of the quality of online health information: systematic literature review of relevant criteria and indicators. *Journal of medical Internet research*, *21*(5), e12522.

Thomas, J., & Harden, A. (2008). Methods for the thematic synthesis of qualitative research in systematic reviews. *BMC medical research methodology*, *8*(1), 1-10.

Tricco AC, Lillie E, Zarin W, et al. (2018). PRISMA extension for scoping reviews (PRISMA-ScR): checklist and explanation. Ann Intern Med 169:467–73.

Authors` contributions:

For peer review only

CW and TMS have contributed to the study conception and design. KMB will prepare the dataset and conduct the analysis. During the analysis, the findings will be presented by KMB and put up for debate between all the authors. KMB will then write the first draft of the protocol manuscript, which will be critically reviewed and refined by CW. Both CW and TMS will comment on the following versions of the manuscript. All authors will read and approve the final version of the manuscript.

Funding statement:

The Ph.D.-project is funded by UiT - the Arctic University of Norway and the Healthy Choices and Social Gradient project, funded by the Research Council of Norway (grant number 289440).

Competing interest`s statement:

The authors declare no competing interests.

For peer review only

BMJ Open

Exploring online health information seeking behavior (OHIS) among young adults. A Scoping Review Protocol

Journal:	*BMJ Open*
Manuscript ID	bmjopen-2023-074894.R1
Article Type:	Protocol
Date Submitted by the Author:	16-Dec-2023
Complete List of Authors:	Bratland, Kristine; UiT Norges arktiske universitet, Department of Community Medicine<br>Wien, Charlotte; University of Southern Denmark, Clinical Research<br>Sandanger, Torkjel; UiT Norges arktiske universitet, Department of Community Medicine
**Primary Subject Heading**:	Public health
Secondary Subject Heading:	Epidemiology, Communication, Research methods
Keywords:	Protocols & guidelines < HEALTH SERVICES ADMINISTRATION & MANAGEMENT, SOCIAL MEDICINE, PUBLIC HEALTH, Decision Making, EPIDEMIOLOGY

SCHOLARONE™
Manuscripts

Exploring online health information seeking behavior (OHIS) among young adults. A Scoping Review Protocol.

Kristine M. Bratland[1], Charlotte Wien[2], Torkjel M. Sandanger[1]
[1]UiT The Arctic university of Norway, [2]University of Southern Denmark, SDU

Correspondence to Kristine M. Bratland: Institutt for Samfunnsmedisin, Hansine Hansens veg 18, 9019 Tromsø, kristine.m.bratland@uit.no, +47 99566701

Charlotte Wien, Clinical Department, University of Southern Denmark SDU, Odense, Denmark
Torkjel Manning Sandanger, Institute of Community Medicine, Tromsø, Norway

Word count - excluding title page, references, figures: 3392.

Keywords: Consumer Health Information, Access to information, Health Communication.

Abstract:

Introduction: In recent years, information technology and social media have experienced unprecedented growth particularly in the Nordic countries. However, there is a noticeable lack of comprehensive understanding regarding the latest research findings on online health information seeking behavior (OHIS) among young adults (>18 – >30 years old). There is a need to conduct an updated review to identify knowledge gaps in where young adults find health information and their user interface (UI) preferences to provide research-based guidance and recommendations to governments, health organizations, and social media platforms on how to facilitate this prominent pattern. The scoping review protocol outlines a study that will systematically map the existing literature on young adults' preferences for digital platforms and platform characteristics in relation to OHIS, enabling the identification of promising areas for further research and the development of more effective interventions to promote healthy and informed choices. Conducting a scoping review is imperative to gain a comprehensive understanding of young adults' OHIS and support the next generation of dissemination that promotes accurate and reliable digital health information.

Methods and analysis: The scoping review will utilize Arksey and O'Malley's methodological framework (PRISMA-ScR) and employ the Citation Pearl Method and the SPIDER model to design the search strategy. To identify relevant literature, three databases will undergo a search: Scopus, Web of Science, and EMBASE. Additionally, a subsidiarily grey literature search will be conducted in Google Scholar. The data charting process will conform to the PRISMA-ScR standard and will be further structured with EndNote. Qualitative and quantitative analyses of the extracted data will be developed using EndNote and Excel.

For peer review only - http://bmjopen.bmj.com/site/about/guidelines.xhtml

Ethics and dissemination: Conducting a scoping review involve secondary data analysis of publicly available sources and does not require an ethical review. The protocol will be published to ensure transparency. The scoping review results will be disseminated through open access peer-reviewed publications, national and international conferences, social media platforms, newspapers, and YouTube to service users and stakeholders.

Strengths and limitations of this study:

- A systematic study conducted following the SPIDER model using EndNote and the PRISMA-ScR checklist.
- Limited to include articles published within the last six years.
- Conducted in an underserved and rapidly developing cross-disciplinary research field.

Introduction

Johnson[1, 2] defined Health Information Seeking Behavior (HISB) as a deliberate attempt to meet a health information need, distinguishing it from passive forms of information acquisition, such as incidental exposure or scanning[3]. The emergence of Online Health Information Seeking Behavior (OHIS) in the late 1990s and early 2000s coinciding with the advent of the internet as a promising source of health information[1, 4, 5]. In the last decade, research on OHIS has grown significantly, driven by the rapid increase in health information available and the shift from an authoritative clinician-patient interaction to shared decision making[6].

The definition of young adults varies across contexts and disciplines. In the United States, young adults are typically defined as individuals between the ages of 18 and 34[7], while in Europe, the age range is often extended to 18-39 [8]. Being a young adult represent unique challenges and opportunities [9], involving the transition from adolescence to adulthood, as well as taking responsibility for their own health. Consequently, the information needs, actions and behaviors differ in adolescents, adults, and seniors, which reflects their behaviors[10].

Exploring the information preferences among young adults is important due to their status as *modern* digital natives[11] individuals who have grown up with digital and technology. As the first generation to grow up with internet and related technologies, young adults have distinct OHIS and preferences compared to older generation[12]. Understanding these differences is crucial for promoting and improving health outcomes among this population[10, 12]. Conducting research on young adults' online health information seeking (OHIS) is essential for identifying gaps in health knowledge, improving health literacy, and promoting health equity[13-15]. Young people are more likely to seek

For peer review only - http://bmjopen.bmj.com/site/about/guidelines.xhtml

out online health information than older adults, and the use of OHIS is increasing among young people[16, 17]. However, we lack an overview on the factors driving these outcomes, and it is still important to gain a deeper understanding of their online health information seeking behavior. This is because young adults may have unique information needs and preferences, and understanding these factors can help healthcare providers and policymakers to develop more effective strategies for promoting health literacy and improving health outcomes among this population.

Understanding how young adults use digital tools to seek and process health information, and how this information shapes their health behaviors is important. Young adults, being heavy users of technology and digital information[16, 17], make a critical audience for online health information[18, 19]. Reviewing the current state of research on OHIS is crucial for informing health communication and intervention strategies due to the potential impact of such information on their health behaviors and outcomes. Such a review is also novel in that it provides a comprehensive synthesis of existing studies, which are often fragmented and lack consistency in conceptualization and measurement of key constructs.

This study aims to address the lack of understanding regarding online health information seeking behavior among young adults. By conducting an updated scoping review, the study will identify knowledge gaps. This protocol advocates for using the scoping review methodology for exploring a broad research topic such as OHIS among young adults[20, 21]..

Methods and analysis

Scooping reviews use a variety of study designs to synthesize evidence and provide a comprehensive summary that informs practice, programs, and policy, while also guiding future research priorities[20-22]. It differs from systematic reviews in its purpose and aims, representing a relatively new approach to synthesizing evidence[23]. Both reviews use rigorous and transparent methods to identify and analyze relevant literature[24]. Where systematic reviews aim to sum up the best available research on a specific question from a relatively smaller number of studies pertaining to a focused research question, the purpose of a scoping review is to map the body of literature on a topic or area[24, 25].

The search strategy is based on the scoping review methodology suggested by Arksey and O'Malley[20] and further developed by Levac, Colquhoun, and O'Brien[21] and Peters et al.[26]. The purpose of a scoping review is to provide an overview of the available research evidence without producing a summary answer to guide clinical decision making[20, 25] and identify gaps in the literature[21, 22].

For peer review only - http://bmjopen.bmj.com/site/about/guidelines.xhtml
For peer review only

While the methodical framework of Arksey and O'Malley[20], further developed by Levac et al.[21], informs the overall conduct of the scoping review, the Preferred Reporting Items for Systematic Reviews and Meta-Analyses (PRISMA) extension for Scoping Reviews'[27] guides the reporting of this protocol and also subsequently structures the reporting of the full review.

Stage 1: Defining the research question

This protocol aims to describe a possible, research-based approach on how to carry out a a scoping review to get an overview of the body of literature on OHIS among young adults. Furthermore, this protocol does not include a research question as it is a preliminary plan for conducting a study. However, a research question has been developed for the study and paper that will be conducted based on this protocol. The overall purpose of the planned scoping review presented in this protocol is:

What does previous studies report on young adults' online health information seeking behavior (OHIS) on health information regarding the choice of digital platform and platform user interface (UI)?

The review is guided by the following objectives:

1) Identifying studies on young adults' online health information seeking behavior
2) Determining which digital platforms young adults tend to search for health information
3) Identifying characteristics in the user interface that apply to young adults' aims and trust
4) Enhancing the characteristics that have an impact.

Stage 2: Identifying relevant literature

In the scoping review on online health information seeking behavior among young adults, a systematic approach is used to choose the search terms. The following steps are taken: A preliminary search is conducted with the support of subject matter experts in the field to identify additional keywords and search terms. Controlled vocabulary (MeSH-terms) and keywords are used to build the first search string, which results in the identification of three relevant papers. Citation Pearl Growing [28] is applied by thoroughly reading the three core papers from the preliminary search and searching for new search terms. The bibliography is revisited to look for relevant references published prior to the core documents that could contain other possible search terms. The search string is revisited and developed to ensure that it captures all the relevant concepts related to the research question. The search string is presented to a new group of experts in the field for feedback, and changes are made accordingly. The initial search string is designed using key elements from the review question, applying the SPIDER

For peer review only - http://bmjopen.bmj.com/site/about/guidelines.xhtml

strategy (Sample, Phenomenon of Interest, Design, Evaluation, Research type), see Table 1. The SPIDER tool is an alternative to the more well-known PICO, better suited when searching for qualitative research papers[29]e, replacing the "O" (Outcome) with the broader term "E" (Evaluation)[29]e.

**Sample**	Young adults in the age range where they make their own choices, meaning over 18, under 30 years old. No limitations in gender.
**Phenomenon of Interest**	Online Health Information Seeking Behavior
**Design**	Published literature of any research design, grey literature
**Evaluation**	"Searching activities" - characteristics, views, experiences, observations, descriptions, surveys, descriptive
**Research type**	Qualitative, quantitative, mixed methods, multi methods peer reviewed studies. Grey literature including third sector and conference proceedings.

Table 1: The SPIDER tool applied to the review question

Table 2 presents the proposed search strategy mapped against the SPIDER tool.

**Sample**	("young adults" OR young OR "early adulthood" OR "early twenties" OR "aged 20") AND
**Phenomenon of Interest**	"Health Information" AND
**Evaluation**	AND (search* OR seek* OR find* OR access* OR retrieve* OR behavior*) AND (internet OR online OR web OR digital* OR media)

Table 2: The proposed search strategy mapped against the SPIDER tool

Boolean operators (AND, OR) are utilized to combine the various search terms, ensuring that relevant articles that meet the inclusion criteria are retrieved. Multiple rounds of trials and errors will be conducted. Through these techniques, the search strings are ensured to be comprehensive and capture the relevant concepts[30] related to online health information seeking behavior among young adults.

When conducting a scoping review, it is important to choose appropriate databases to ensure that all relevant studies are captured[20, 26]. To identify relevant articles for the scoping review on online health information seeking behavior among young adults, a comprehensive search is conducted on three electronic databases: EMBASE, Web of Science, and SCOPUS. These three databases are commonly used in health research, and each has its own strengths and weaknesses[31, 32]. By searching multiple databases, the risk of missing important studies that may be indexed in one database but not in another is reduced. These databases also have advanced search capabilities that allow for refinement of the search strategy and retrieval of only the most relevant studies[32]. They are widely used in health research, which means that the review can be compared and contextualized with other studies that have used similar methods and data sources.

After identifying the relevant articles from the databases, the citation pearl method will be used to identify additional relevant studies. This method involves examining the reference lists of the selected articles to identify any other relevant studies that may not have been captured in the initial search[33]. A forward citation search will also be conducted to identify any new articles that cite the selected

For peer review only

For peer review only - http://bmjopen.bmj.com/site/about/guidelines.xhtml

articles since their publication. This will help to identify newer studies that may not have been captured in the initial search or the citation pearl search. The citation pearl method is a commonly used technique in systematic reviews and scoping reviews to ensure that all relevant studies are included in the analysis[27]. It is particularly useful when searching for grey literature or when there are no standard indexing terms to identify relevant articles. By using this method, additional studies that meet the inclusion criteria will be identified, ensuring that the scoping review is comprehensive and robust. After the advanced search, a search will be conducted in Google Scholar using the advanced search feature to detect grey literature. Specific search filters will be applied, such as limiting results to conference proceedings, reports, theses, dissertations, and other non-peer-reviewed sources. Furthermore, an advanced search will be conducted for grey literature using Google Scholar to ensure that no relevant studies are missed[30]. By using Google Scholar, our search for grey literature will be broadened beyond traditional academic sources to identify additional studies on online health information seeking behavior among young adults. A combination of keywords and advanced search techniques will be employed to retrieve relevant grey literature[33]. This approach will help identify a range of studies that may have been missed in the initial database search. By using traditional academic databases and Google Scholar to search for grey literature, the scoping review on online health information seeking behavior among young adults will be made as comprehensive as possible, including all available evidence on the topic.

Stage 3: Article selection

To identify papers about OHIS behavior in young people, a set of inclusion and exclusion criteria is developed. The aspects are presented in table 3. The following will be the criteria for inclusion:

1) The topic of the article must be health-related, or encompassing topics e.g., mental health, diet, or nutrition.
2) The study must target young adults or specifically address the population of young adults (18 – 30 years old) or make explicit, equivalent assertions. Although the search strategies indicate two commonly accepted lower age boundaries, “early twenties” and “aged 20” to identify young adults above teens, it will not exclude other ways to describe the population.
3) Describe OHIS activities or individual preferences or assessments, e.g., general OHIS, search strategies, choice of health information sources, or use of health information.
4) The paper must be published in 2017 or later, to make the results applicable to today's digital landscape.

For peer review only - http://bmjopen.bmj.com/site/about/guidelines.xhtml

The exclusion criteria are as follows:

1) Studies that focus on the use of general information and communication technology or behavior change, instead of focusing on health information will be excluded.
2) Papers where age is a determinant in studying the OHIS of the overall population will be excluded, as it is apparent that age has an influence on individual OHIS behavior. Abstracts, posters, op-eds, or letters will be excluded as they are not full papers.
3) Papers that are not published in a peer-reviewed journal or in the proceedings of a conference will be excluded.
4) Papers written in languages other than English, Norwegian, Swedish, or Danish will be excluded.

Criterion	Definition
**Population**	Population of young adults (>18 and ≤30), or explicit address this part of the population. Surveys that also screened children or adults will be included if the procedure for young adults is described.
**Screening procedure**	Peer reviewed publications on a health-related topic with the aim of describing, elaborate, systematize, and/or quantifying online health information seeking behavior, use of internet, search engines, browsers, web 2.0 or 3.0, social media or other online platforms retrieving information.
**OHIS definitions**	A qualitative or quantitative description of an active effort to obtain health-related information about a specific topic, using any digital sources to obtain desired information, that occurs outside of routine patterns of interpersonal communication or media use[6, 18, 34].
**Platform**	Interested in determining what digital platforms young adults tend to use when searching for health information
**Characteristics**	Interested in identifying traits, characteristics, or interface on these digital platforms that young adults prefer, enjoy, or has a significant meaning for those using the exact platform
**Setting**	Primarily observational studies or self-reported studies from the global north. However, may include studies developed in other parts of the world if the methodology employed is subsequently.

Table 3: Inclusion and exclusion criteria

When the search is conducted and the articles are retrieved in EndNote, the next step will involve screening them for eligibility based on established criteria. A three-stage screening process will be performed, where researchers will first remove duplicates. The next step will be to screen titles and abstracts to exclude obviously irrelevant articles. This will be followed by a full-text screening of the

For peer review only - http://bmjopen.bmj.com/site/about/guidelines.xhtml

remaining articles to determine whether they meet the inclusion criteria. Additionally, EndNote will be used as a citation management software to facilitate the screening process and keep track of the articles throughout the review. Each step of the process will be filed in separate EndNote Libraries. The reasons for excluding articles will be documented, and the progress will be made visible by presenting a PRISMA flow diagram, which will include searches of the databases, registers, and other sources. This will ensure transparency and reproducibility of the review process.

Once the search is conducted and the articles are retrieved and stored in a labeled EndNote-library, the next step will involve screening them for eligibility based on the established criteria. This will involve a two-stage screening process, where titles and abstracts will be first screened to exclude obviously irrelevant articles, followed by a full-text screening of the remaining articles to determine whether they meet the inclusion criteria. The results in each step will be stored in new separated EndNote libraries. This will be important to document the process of excluding articles, to ensure transparency and reproducibility of the review process, and will make the scoping review comprehensive, relevant, and reliable.

Stage 4: Data charting

To record and synthesize the extracted data, a data charting form will be used. The suggested form is presented in table 4, The form will help to organize the data into categories and subcategories and identify gaps in the literature. Descriptive statistics such as frequency and percentage will be used to summarize the extracted data. The findings of the review will help to identify current trends, patterns, and factors influencing young adults' OHIS behavior, including the benefits and risks associated with OHIS. The results will also inform the development of strategies to improve the quality and accessibility of online health information for young adults.

Variable	Definition
First author	The last name of the papers first author
Year	The year the paper was published
Title	Title of the article
Journal	Name of the journal
Nationality	Nationality of the publisher/university
Country	Country/nationalities of the population
Sample Size	The number of participants, informants, or observations
Population	Group, sex, age

For peer review only - http://bmjopen.bmj.com/site/about/guidelines.xhtml

Method	Type of data and/or tools: Qualitative (survey, interview, administrative data, observation, focus group) quantitative (check list, self-report, chart reviews, electronic health record, validated questionnaire,
Results	The main results or findings
Other notes	Additional information about the paper

Table 4: Variables and definitions for data chartering

Stage 5: Collating, summarizing, and reporting the results

EndNote and Excel will be used to arrange and scrutinize the data extracted from the full-text articles. The goal is to produce both quantitative and qualitative data while analyzing the articles comprehensively to evaluate them and achieve the objectives of the review. Excel will be used to analyze the data generated, and following the PRISMA guidelines[27], assessments of the qualitative and quantitative data will be integrated to present the results. Findings will be tabulated by identifying:

1) Where young adults report finding health information
2) Choice of digital platform
3) Preferences regarding user interface

Patient and Public Involvement

This study is a scoping review, which involves searching for and mapping the existing literature on a particular topic. As such, there will be no direct involvement of the public in the study beyond the dissemination and publication of the results.

Expected limitations and strengths of review finding

The upcoming scoping review on online health information seeking behavior among young adults will aim to identify research gaps, map, and synthesize evidence, and summarize findings using a transparent and systematic approach. The study will focus on identifying which digital platforms young adults tend to search for health information, as well as characteristics in the user interface that young adults trust and find useful when seeking health information online. The scoping review will be conducted using the Arksey and O'Malley[20] framework and the PICO model[35] to develop a search strategy, while Excel will be used to chart and organize the data, and the PRISMA-ScR checklist[27] will guide the reporting of findings. To ensure the quality of the scoping review, the search will be limited to studies published in peer-reviewed journals. However, the heterogeneity of the included studies

For peer review only - http://bmjopen.bmj.com/site/about/guidelines.xhtml

For peer review only

will be acknowledged to limit the ability to provide a quantitative synthesis of the data, such as a meta-analysis. Instead, a focus will be made on a qualitative synthesis of the evidence, which will provide a comprehensive overview of the research in the field. By synthesizing the evidence, gaps in the literature will be identified, and insights into the characteristics of digital platforms that are most effective for young adults when seeking health information online will be provided. The findings will be reported in a peer-reviewed journal, following the scoping review reporting standards (PRISMA-ScR) and disseminated widely to stakeholder groups using innovative media approaches.

Ethics and dissemination

Ethical considerations will remain essential in all health research, including scoping reviews. In Norway, the ethical principles for medical research are set out in the Norwegian Health Research Act[36], which emphasizes the importance of protecting the rights and privacy of research participants. As this scoping review will not involve human research participants, it will not be necessary to obtain informed consent, protect anonymity, or receive institutional board approval. However, we will still ensure the integrity of the research and respect for all potential stakeholders by following established guidelines for conducting scoping reviews, such as the PRISMA-ScR checklist[27] and the Arksey and O'Malley[20] framework. The findings will be disseminated through various channels, including open access peer-reviewed journal publications, national and international conference presentations, academic social media platforms, newspaper articles, and blog posts. By sharing our results widely, the aim will be to inform and engage a diverse audience, including individuals affiliated with our institutions and professional associations.

Authors` contributions:

This protocol was developed by KMB. CW and TMS have contributed to the study conception and design. KMB developed the search string in collaboration with two senior librarians from UiT and SDU. The search string presented has been discussed between KMB, CW and TMS during the process. Conducting the final retrieving of literature, KMB will prepare the dataset and conduct the analysis. During the analysis, the findings will be presented by KMB and put up for debate between KMB, CW and TMS. KMB will then write the first draft of the article manuscript, which will be critically reviewed and refined by CW. Both CW and TMS will comment on the following versions of the manuscript. KMB, CW, TMS will read and approve the final version of the manuscript before submission.

For peer review only - http://bmjopen.bmj.com/site/about/guidelines.xhtml

Competing interest`s statement:

The authors declare no competing interests.

Funding statement:

The Ph.D.-project is funded by UiT - the Arctic University of Norway and the Healthy Choices and Social Gradient project, funded by the Research Council of Norway (grant number 289440).

For peer review only

For peer review only - http://bmjopen.bmj.com/site/about/guidelines.xhtml

References

1. Galarce, E.M., S. Ramanadhan, and K. Viswanath, *11 HEALTH INFORMATION SEEKING.* The Routledge handbook of health communication, 2011.
2. Johnson, J.D., *Cancer-related information seeking.* (No Title), 1997.
3. Shim, M., B. Kelly, and R. Hornik, *Cancer information scanning and seeking behavior is associated with knowledge, lifestyle choices, and screening.* Journal of Health Communication, 2006. **11**(S1): p. 157-172.
4. Eysenbach, G., et al., *Empirical studies assessing the quality of health information for consumers on the world wide web: a systematic review.* Jama, 2002. **287**(20): p. 2691-2700.
5. Dutta-Bergman, M.J., *Primary sources of health information: Comparisons in the domain of health attitudes, health cognitions, and health behaviors.* Health communication, 2004. **16**(3): p. 273-288.
6. Lewis, N., N. Shekter-Porat, and H. Nasir, *Health Information Seeking.* The Routledge Handbook of Health Communication, 2021: p. 399-411.
7. Data, S.E.C. *Young adults*. Available from: https://census.socialexplorer.com/young-adults/#/.
8. Eurostat., *Young people - Statistics on Europe.* . 2021.
9. Arnett, J.J., *Emerging adulthood: A theory of development from the late teens through the twenties.* American psychologist, 2000. **55**(5): p. 469.
10. Gray, N.J., et al., *Health information-seeking behaviour in adolescence: the place of the internet.* Soc Sci Med, 2005. **60**(7): p. 1467-78.
11. Prensky, M., *Digital natives, digital immigrants part 2: Do they really think differently?* On the horizon, 2001. **9**(6): p. 1-6.
12. Kim, H. and B. Xie, *Health literacy in the eHealth era: a systematic review of the literature.* Patient education and counseling, 2017. **100**(6): p. 1073-1082.
13. Azzopardi-Muscat, N. and K. Sørensen, *Towards an equitable digital public health era: promoting equity through a health literacy perspective.* European journal of public health, 2019. **29**(Supplement_3): p. 13-17.
14. Sun, Y., et al., *Consumer evaluation of the quality of online health information: systematic literature review of relevant criteria and indicators.* Journal of medical Internet research, 2019. **21**(5): p. e12522.
15. Diviani, N., et al., *Low health literacy and evaluation of online health information: a systematic review of the literature.* Journal of medical Internet research, 2015. **17**(5): p. e112.
16. Wang, X., J. Shi, and H. Kong, *Online health information seeking: a review and meta-analysis.* Health Communication, 2021. **36**(10): p. 1163-1175.
17. Jia, X., Y. Pang, and L.S. Liu. *Online health information seeking behavior: a systematic review*. in *Healthcare*. 2021. MDPI.
18. Lewis, T., *Seeking health information on the internet: Lifestyle choice or bad attack of cyberchondria?* Media, Culture & Society, 2006. **28**(4): p. 521-539.
19. Jensen, M., et al., *Young adolescents' digital technology use and mental health symptoms: Little evidence of longitudinal or daily linkages.* Clinical Psychological Science, 2019. **7**(6): p. 1416-1433.
20. Arksey, H. and L. O'Malley, *Scoping studies: towards a methodological framework.* International journal of social research methodology, 2005. **8**(1): p. 19-32.
21. Levac, D., H. Colquhoun, and K.K. O'Brien, *Scoping studies: advancing the methodology.* Implementation science, 2010. **5**: p. 1-9.
22. Thomas, J. and A. Harden, *Methods for the thematic synthesis of qualitative research in systematic reviews.* BMC medical research methodology, 2008. **8**(1): p. 1-10.

For peer review only - http://bmjopen.bmj.com/site/about/guidelines.xhtml

23. Sucharew, H. and M. Macaluso, *Methods for research evidence synthesis: The scoping review approach.* Journal of hospital medicine, 2019. **14**(7): p. 416-418.
24. Library., W.C.M., *Scoping reviews.*
25. Munn, Z., et al., *The development of software to support multiple systematic review types: the Joanna Briggs Institute System for the Unified Management, Assessment and Review of Information (JBI SUMARI).* JBI evidence implementation, 2019. **17**(1): p. 36-43.
26. Peters, M.D., et al., *Guidance for conducting systematic scoping reviews.* JBI Evidence Implementation, 2015. **13**(3): p. 141-146.
27. Tricco, A.C., et al., *PRISMA extension for scoping reviews (PRISMA-ScR): checklist and explanation.* Annals of internal medicine, 2018. **169**(7): p. 467-473.
28. Ramer, S.L., *Site-ation pearl growing: methods and librarianship history and theory.* Journal of the Medical Library Association, 2005. **93**(3): p. 397.
29. Cooke, A., D. Smith, and A. Booth, *Beyond PICO: the SPIDER tool for qualitative evidence synthesis.* Qualitative health research, 2012. **22**(10): p. 1435-1443.
30. Adams, R.J., P. Smart, and A.S. Huff, *Shades of grey: guidelines for working with the grey literature in systematic reviews for management and organizational studies.* International Journal of Management Reviews, 2017. **19**(4): p. 432-454.
31. Stapleton, J., C. Carter, and L. Bredahl, *Developing systematic search methods for the library literature: Methods and analysis.* The Journal of Academic Librarianship, 2020. **46**(5): p. 102190.
32. Bramer, W.M., D. Giustini, and B.M. Kramer, *Comparing the coverage, recall, and precision of searches for 120 systematic reviews in Embase, MEDLINE, and Google Scholar: a prospective study.* Systematic reviews, 2016. **5**: p. 1-7.
33. Bramer, W.M., et al., *Searching Embase and MEDLINE by using only major descriptors or title and abstract fields: a prospective exploratory study.* Systematic Reviews, 2018. **7**: p. 1-8.
34. Case, D.O. and L.M. Given, *Looking for information: A survey of research on information seeking, needs, and behavior.* 2016.
35. Frandsen, T.F., et al., *Using the full PICO model as a search tool for systematic reviews resulted in lower recall for some PICO elements.* Journal of clinical epidemiology, 2020. **127**: p. 69-75.
36. https://lovdata.no/dokument/NL/lov/2008-06-20-44, H., *Lov om medisinsk og helsefaglig forskning (helseforskningsloven). LOV-2008-06-20-44.* 2009.
37. Grajales, F.J., 3rd, et al., *Social media: a review and tutorial of applications in medicine and health care.* Journal of medical Internet research, 2014. **16**(2): p. e13-e13.

For peer review only

For peer review only - http://bmjopen.bmj.com/site/about/guidelines.xhtml
